# Infrequent Histopathologic Subtypes of Oral Squamous Cell Carcinoma: A Case Series with Emphasis on Histopathologic Characteristics 

**DOI:** 10.30476/dentjods.2025.103223.2435

**Published:** 2025-06-01

**Authors:** Saede Atarbashi-Moghadam, Ali Lotfi, Leyla Roghanizadeh, Seyed Sepehr Mirebeigi Jamasbi, Zeinab Akbarzadeh Fathabadi

**Affiliations:** 1 Dept. of Oral and Maxillofacial Pathology, School of Dentistry, Shahid Beheshti University of Medical Sciences, Tehran, Iran.; 2 Researcher, Iranian Center for Endodontic Research, Research Institute for Dental Sciences, Shahid Beheshti University of Medical Sciences, Tehran, Iran.; 3 Dental Student, Student Research Center, School of Dentistry, Shahid Beheshti University of Medical Sciences, Tehran, Iran.

**Keywords:** Basaloid Squamous Cell Carcinoma, Carcinoma Cuniculatum, Spindle Cell Carcinoma, Squamous Cell Carcinoma

## Abstract

Squamous cell carcinoma (SCC) is the most common oral malignancy. Conventional types are classified as well-, moderately- and poorly differentiated, which are usually easy to diagnose microscopically. Still, uncommon variants such as basaloid, verrucous, spindle cell, papillary, adenosquamous, acantholytic, cuniculatum, clear cell, and pigmented SCC make a diagnostic challenge for pathologists. This report presents four rare cases of oral SCC with histopathologic diagnosis of spindle cell carcinoma, carcinoma cuniculatum, papillary SCC, and basaloid SCC focusing on microscopic characteristics and differential diagnosis. The apprehensive knowledge about the unique histopathologic features of these uncommon variants is crucial to avoid their misdiagnoses and provide appropriate treatment.

## Introduction

Squamous cell carcinoma (SCC) is the most predominant cancer of the oral region with different microscopic subtypes [ 1 - 2 ]. Oral cancers are currently a global concern because of their high incidence and low five-year survival rates [ 3 ]. In addition, they may lead to functional and aesthetic problems such as speaking and swallowing [ 4 ].

Many risk factors including alcohol and tobacco, geographic variation, genetic predisposition, diets, immune status, oncogenic viruses, radiation, poor oral hygiene, environmental factors, and obesity, are involved in oral cancer. Moreover, diabetes and iron deficiency may increase the risk of cancers of the oral cavity [ 5 ]. Conventional oral SCCs are classified histologically as well- (grade 1), moderately- (grade 2), and poorly differentiated (grade 3) according to the amount of keratinization, cellular and nuclear atypia, and mitotic activity. Well and moderately differentiated SCC can be categorized together as low grade and poorly differentiated and undifferentiated tumors as high grade [ 1 ]. Other subtypes were described in the literature as basaloid, verrucous, spindle cell, papillary, adenosquamous, acantholytic, cuniculatum [ 2 , 6 - 7 ], and glycogen-rich clear cell variants [ 6 ]. Furthermore, pigmented and intra-osseous SCCs are also reported . 

Although conventional types are easy to diagnose histopathologically, infrequent variants of SCC can be a diagnostic challenge for pathologists. Thus, this study aims to report such cases focusing on microscopic characteristics and differential diagnosis. Acquaintance with these features is essential for oral pathologists. 

## Case Series

### Case Presentation 1 

**Figure 1:**
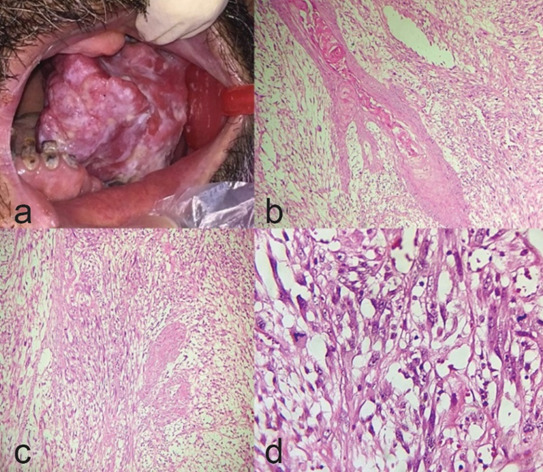
Spindle cell SCC; **a:** A large pedunculated polypoid mass of the alveolar ridge of maxilla; **b:** Spindle cell proliferation and dysplastic superficial epithelium (100×, hematoxylin and eosin staining (H & E)); **c:** Spindle cell proliferation (100×, H & E); **d:** Pleomorphic spindle cells with mitosis (400×, H & E)

**Figure 2:**
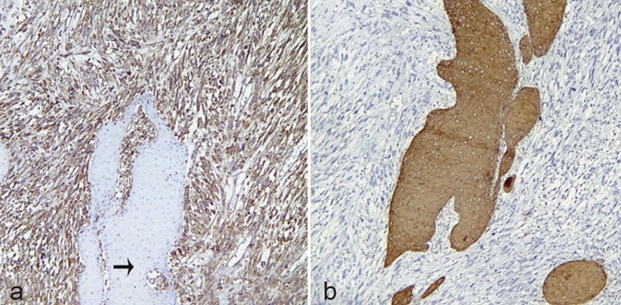
Spindle cell SCC; **a:** Positive cytoplasmic immunoreactivity of spindle cells for vimentin and absence of staining of the epithelial island (black arrow) (100×, IHC); **b:** Positive cytoplasmic immuno-reactivity of epithelial islands for AE1/AE3 (100×, IHC)

A 65-year-old male with poor socioeconomic status was referred to a private dental clinic (Tehran, Iran) for evaluation of a huge non-tender polypoid ulcerated and erythematous pedunculated mass attached to the crest of the posterior area of the edentulous maxillary ridge which had been present for an unknown duration. The mass was soft to elastic in consistency measuring 5.5×4 cm (Figure 1a). The lesion interfered with eating, talking, and closing the mouth. There was no history of previous trauma and he was a cigarette smoker. There was no cervical lymphadenopathy on clinical examination. The lesion was removed with excisional biopsy with a provisional diagnosis of soft tissue tumor and reactive lesion. Microscopic sections revealed a malignant neoplasm covered by dysplastic surface epithelium (severe dysplasia) in conjunction with fascicles of invasive spindle cell elements with many mitotic figures and scattered islands of dysplastic squamous cells (Figures 1b-d). The neoplastic cells showed diffuse immunoreactivity for vimentin. CK (AE1/AE3) was positive in epithelium, squamous islands, and scattered mesenchymal cells (Figures 2a-b). These findings were consistent with the spindle cell carcinoma (SpCC) (polypoid or sarcomatoid SCC) diagnosis. He refused any other treatment. 

### Case Presentation 2 

**Figure 3:**
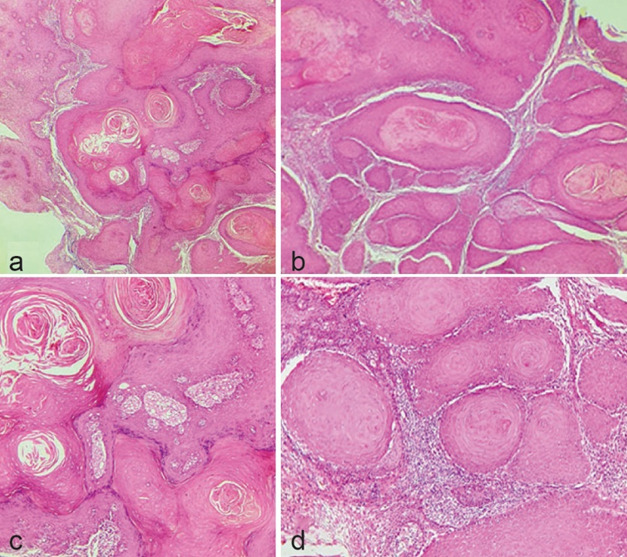
Carcinoma cuniculatum; **a:** “Nest-like” epithelium filled with keratinaceous material (40×, H & E); **b-d:** Epithelial cells surrounding keratinaceous material show significant mild atypia (100×, H & E)

A 57-year-old woman was referred to the clinic mentioned above with an exophytic erythematous mass with uneven granular surface on the left buccal surface of maxillary gingiva in the alignment of teeth #21, 22, and 23 measuring 1.5×1cm. The patient has noticed the changes in this area for 4 years, but the prominent changes and mass formation occurred 7-8 months ago. She was not a smoker. The panoramic radiograph was unremarkable. There was no cervical lymphadenopathy on clinical examination. An incisional biopsy was done with a provisional diagnosis of granulomatous inflammation and SCC. The underlying bone was intact. Histopathologic sections showed keratin-filled invaginations that burrow deep into the stroma. The epithelial cells showed mild atypia. Infiltration of inflammatory cells was also evident. The diagnosis of carcinoma cuniculatum (CC) was made based on clinical and microscopic features and she was referred for excisional biopsy (Figures 3a-d).

### Case Presentation 3

**Figure 4:**
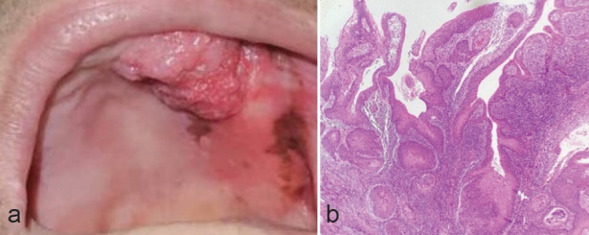
Papillary SCC; **a:** Exophytic papillary mass and pigmented areas of the left maxillary alveolar mucosa and palate; **b:** Multiple papillary projections with fibrovascular cores, in addition to prominent dysplastic changes and severe inflammation can be observed (100×, H & E)

A 75-year-old woman was referred to the clinic mentioned earlier with large painful exophytic papillary mass and pigmented areas of the left maxillary alveolar mucosa and palate with at least a period of 4 months. The lesion was pink with papillary/verrucous projections and firm consistency measuring 2×1cm. There were also three pigmented macules in the vicinity of the lesion (Figure 4a). She was not a smoker. There was no cervical lymphadenopathy on clinical examination. A provisional diagnosis of papillary SCC (PSCC) and melanoma was made, and an incisional biopsy was performed under local anesthesia. Microscopic examination displayed multiple papillary projections with fibro-vascular cores. Prominent dysplastic changes such as cellular pleomorphism, hyperchromatism, mitotic figures, keratin pearls, and microabscesses were also evident (Figure 4b). The pigmented part was similar to a melanotic macule and no melanoma changes were seen. According to the information above, the diagnosis of PSCC was made. The patient was referred for excisional surgery. 

### Case Presentation 4

**Figure 5:**
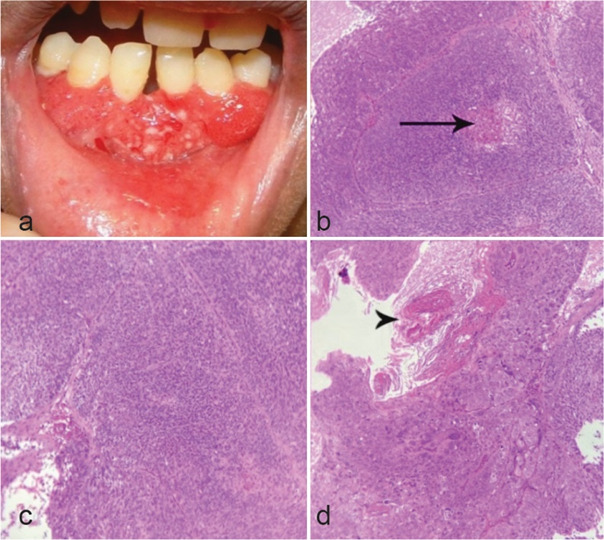
Basaloid SCC; **a:** A large erythematous proliferative mass with granular surface; **b:** Basaloid islands with comedonecrosis (black arrow) (100×, H & E); **c:** Basaloid islands (100×, H & E); **d:** Keratin pearl formation (black arrow head) (100×, H & E)

A 29-year-old woman was referred to the aforementioned clinic with a large painful proliferative and erythematous mass with a granular surface in the anterior gingiva extended to the labial mucosa (Figure 5a). A reticular pattern of skin hyperpigmentation was seen, affecting the face, neck, and upper chest. However, the genetic analysis did not reveal any specific genodermatosis disorder. An incisional biopsy was performed with a provisional diagnosis of SCC. Microscopic sections displayed a highly malignant epithelial neoplasm composed of solid islands with peripheral basaloid cells and squamous cells in the center. Keratin pearl formation, comedonecrosis, and severe pleomorphism with many mitotic figures were evident (Figures 5b-d). The diagnosis of basaloid SCC (BSCC) was made and she was referred for excisional biopsy and further treatment.

## Discussion

Identification of uncommon histopathological features of SCC helps straightforward diagnosis and timely treatment of patients, which subsequently can be influential in saving patients’ lives. In this case series study, these infrequent subtypes were investigated and discussed.

SpCC/ polypoid SCC/ sarcomatoid carcinoma is an uncommon aggressive subtype of SCC described by dysplastic surface epithelium in combination with an invasive spindle cell component [ 2 , 10 ]. The predisposing risk factors include tobacco and alcohol use [ 6 ], and previous radiotherapy of the head and neck area has also been mentioned [ 11 ]. It tends to arise in older adults with male predilection . SpCC classically shows an exophytic polypoid mass with an ulcerated surface to an infiltrative ulcer, occurring mostly in the alveolar ridge. Pedunculated appearance is also seen [ 13 ]. The presented case (#1) also showed a pedunculated mass with a polypoid surface. This clinical profile varies from conventional oral SCC, which mainly occurs as an ulcer in the lateral border of the tongue and floor of the mouth. Immunohistochemistry (IHC) is an important tool for diagnosis confirmation, particularly when the carcinomatous element is not seen in the biopsy. However, the diagnosis of most variants of SCC is based on the hematoxylin and eosin (H & E) staining and IHC has limited diagnostic value [ 14 - 15 ]. Table 1 demonstrates some IHC descriptions of rare SCC variants. Typically, Vimentin and Panactin are positive in the spindle cell component, and expression of α -smooth muscle actin (α-SMA), desmin, and S-100 protein is variable . Moreover, P63 is a useful marker for detecting epithelial nature [ 10 ]. It has been described that almost 20% of extra-oral SpCC can be negative for AE1⁄AE3 in both neoplastic elements [ 6 ]. Bony or cartilaginous components within the tumor may be seen [ 12 ]. Differential diagnosis of sarcomatoid carcinoma comprises other malignant mesenchymal tumors such as spindle cell melanoma, leiomyosarcoma, and myoepithelial carcinoma . In the current case (#1), despite the scattered expression of cytokeratin in spindle cells, the presence of significant dysplasia of the epithelial surface and tumoral islands was completely indicative of SpCC. It has been noted that it shows a similar prognosis to conventional SCC [ 10 ]. 

**Table 1:** Immunohistochemistry (IHC) assay description of the histopathologic variants of squamous cell carcinoma (SCC)

Histopathologic variant	IHC description
Spindle cell SCC	● Vimentin and Panactin are positive in the spindle cell component
	● Expression of α -smooth muscle actin (α-SMA), desmin, and S-100 protein is variable
	● P63 is a useful marker for detecting the epithelial nature of lesion
	● Almost 20% of extra-oral SpCC can be negative for AE ⁄AE3 in both neoplastic elements
Carcinoma cuniculatum, verrucous carcinoma, and papillary SCC	● IHC has limited diagnostic value
	● Low Ki67 expression (<5–15%) has been described in the basal and suprabasal cells of the neoplasm
	● A significant higher ki-67 expression is seen in papillary SCC versus verrucous carcinoma but there is no significant difference between papillary SCC and conventional SCC
Basaloid SCC	● A specific IHC pattern has not been reported to distinguish basaloid variant from other tumors, and its diagnosis is based on H&E staining
	● Staining pattern of P63 is useful to distinguish basaloid SCC from adenoid cystic carcinoma
	● Cocktails of keratins including Cam 5.2, pankeratin AE/AE3 and CK7have been suggested as useful markers
Pigmented SCC	● IHC has limited diagnostic value
Clear cell SCC	● Clear cells of this neoplasm show immuno-reactivity with AE1/AE3, CK5/6, and p63
	● The cells are negative for αSMA, S100, HMB45, Melan-A, CD10, and p16
Acantholytic SCC	● The presence of intracytoplasmic mucin leads to the exclusion of acantholytic SCC and increases the probability of diagnosis of mucoepidermoid carcinoma or adenosquamous carcinoma
	● To rule out angiosarcoma, CD31, CD34, and FLI-1 are required

Oral CC is an infrequent subtype of SCC. Because patients show specific clinical and microscopic characteristics, the 2017 World Health Organization (WHO) classification of head and neck tumors categorized oral CC as an independent subtype of SCC [ 7 ]. CC includes about 2.7% of oral SCCs [ 16 ]. There is no sex predilection and more prevalent in middle-aged and elderly [ 17 ]. The most common location is the gingiva and tongue respectively [ 18 ]. Cutaneous CC shows a clear relationship with human papillomavirus (HPV) infection, but no such association has been mentioned in oral CC. Furthermore, no document has confirmed an association between tobacco and alcohol consumption and oral CC [ 7 ].

Due to the rarity of this lesion, definitive diagnosis is challenging and it is included in the microscopic differential diagnosis of SCC and verrucous carcinoma (VC) [ 7 ]. CC reveals an invasive growth of “nestlike” epithelium filled with keratinaceous material. The growth pattern of the islands has been described as rabbit burrows (cuniculi) [ 15 ]. Epithelial cells surrounding keratinaceous material show significant differentiation and may display only mild atypia and microabscess formation . A prominent inflammatory cells infiltration including lymphocytes, plasma cells, eosinophils, and neutrophils is seen [ 7 ]. Moreover, lack of pronounced dysplasia on microscopy may lead to misdiagnosis of pseudo-epitheliomatous hyperplasia [ 20 ]. Pseudo-epitheliomatous hyperplasia displays a tongue-like epithelial extension of the epithelium in underlying connective tissue without cellular atypia and differentiating it from invasive SCC may be difficult, particularly in small biopsies, inappropriate orientation, and dense inflammatory infiltration [ 21 ]. Most patients with CC show a good prognosis and only a small percentage of patients may have metastasis to lymph nodes [ 7 ]. 

VC is typically exophytic, and the infiltrative front can be described as broad pushing, which may normally occupy only the lamina propria and cause local damage. Contrary to these features, CC exhibits deep and often complex branching keratin-filled epithelial tunnels, which may deeply infiltrate into the submucosal layers and bony tissues. A good clinical, radiographic, and histopathologic correlation is required for a definite diagnosis. Well-differentiated SCC shows cytologic atypia, many mitoses, and keratin pearls that are frequently small, which is completely different from CC . The treatment of choice is complete surgical excision. Lymph node involvement is rare, and lymph node dissection is done only when the CT results propose metastasis [ 22 ]. The prognosis is better than conventional SCC [ 7, 15]. 

PSCC is another rare variant of SCC of the upper aerodigestive tract [ 23 ]. The most common location in oral cavity is the buccal mucosa and gingiva with a chief complaint of exophytic mass with or without pain [ 24 ]. There is a male predilection and most patients are more than 50 years old. Smoking, alcohol consumption, and immunosuppression, and HPV infection have been proposed as predisposing factors [ 25 ].

PSCC occurs in two forms. One of them is more common in the oropharynx and larynx, while the other, is seen in the anterior oral cavity as a keratotic or non-keratotic warty mass. The first variant displays a papillary exophytic squamous epithelial proliferation with full-thickness dysplasia of the epithelium. Most of them are nonkeratinized and stromal invasion is seen in approximately 40% of cases. The anterior oral cavity lesion reveals an exophytic and endophytic squamous proliferation with variable percentages of keratin and cellular atypia; they frequently show keratin pearls and microabscesses at the tips of rete ridges [ 26 ]. The differential diagnoses include VC and CC. VC displays parakeratosis, bulbous frond-like rete ridges, and scarce to minimal epithelial atypia without blurring of the epithelium-stroma interface. CC, typically demonstrates a smooth rather than warty surface and complex, arborizing keratin-filled burrows in the stroma [ 15 , 26 ]. PSCC shows better prognosis than conventional SCC [ 23 - 24 ]. 

BSCC is an infrequent and high-grade variant of SCC and the most common site is the larynx, hypopharynx, oropharynx, epiglottis, and the base of the tongue. The main risk factors are heavy tobacco and alcohol consumption [ 1 , 14 ]. There is a male predilection between 60 and 80 years old. BSCCs show ﬂat or somewhat elevated tumors, often with central ulceration. The most common growth pattern consists of solid nests with a basaloid cell population at the periphery and squamous in the center [ 14 ]. It can display lobular, cord-like, cribriform, tubular, and glandular-like patterns. Cystic spaces in the central portion of nests and comedonecrosis are also seen [ 1 ]. The microscopic differential diagnosis comprises a solid variant of adenoid cystic carcinoma. However, the latter does not demonstrate any propensity towards squamous differentiation. In addition, it has myoepithelial cells and lacks pleomorphic atypical cells, mitosis, and comedonecrosis. Small cell neuroendocrine carcinoma may show some similarities but neuroendocrine markers and the ‘‘dot-like’’ immunostaining with keratins are helpful. Some BSCCs display cysts or pseudo-adenoid structures and may resemble adenosquamous carcinoma, but the latter is mucin-positive and shows true ductulo-acinar differentiation [ 14 ]. The rare intra-osseous variant of the BSCC has also been reported [ 27 ]. Second primary neoplasms have been described in BSCC patients. The higher aggressiveness of BSCC compared to conventional SCC is a challenging issue [ 14 ]. Although some have stated that it shows a worse prognosis and has a higher tendency to local invasion and metastasis [ 28 ].

Pigmented melanocytes have been reported in various non-melanocytic neoplasms such as neuroendocrine carcinoma, and salivary gland tumors [ 29 ]. SCC with melanin pigmentation is an infrequent entity and has been described in various regions such as the skin, uterine cervix, and conjunctiva. It shows similar microscopic features of typical SCC; in addition to the presence of non neoplastic melanocytes within the lesion [ 8 ]. Tran *et al*. [ 8 ] suggested that pigmented SCC has a better prognosis. Increasing melanin production with its antioxidant properties may be effective in the protective immune response [ 8 ].

CCSCC is a rare microscopic subtype of SCC with aggressive behavior. It shows cells with abundant clear cytoplasm along with enlarged and centrally placed round nuclei. The ratio of clear cells to diagnose CCSCC should be more than 25%. It is essential to distinguish CCSCC from other neoplasms containing clear cells. Clear cell mucoepidermoid carcinoma is mucicarmine positive. Clear cell myoepithelial carcinoma shows myoepithelial markers such as α-SMA [ 30 ]. Squamous differentiation and connection with surface oral epithelium excluded hyalinizing clear cell carcinoma and clear cell odontogenic carcinoma. Melanoma shows immuno-reactivity with S100, Melan-A, and HMB-45. Metastatic renal cell carcinoma is CD10 positive. According to the limited number of CCSCC patients described in the oral region, CCSCC is an aggressive type of SCC with a poor prognosis [ 30 ].

Acantholytic SCC is considered a typical SCC in combination with pseudoglandular structures, dyskeratotic cells, and prominent acantholysis within tumor islands. Loss of desmosomal junctions leads to these microscopic changes and it probably has the same prognosis as conventional SCC [ 31 ]. The appearance of acantholytic cells may be bizarre, large, or multinucleated, and variable mitotic numbers are present. It should be noted that the presence of intracytoplasmic mucin leads to the exclusion of acantholytic SCC and increases the probability of diagnosis of mucoepidermoid carcinoma or adenosquamous carcinoma. To rule out angiosarcoma, CD31, CD34, and FLI-1 are required [ 32 ]. There is no evidence that oral acantholytic SCC is more aggressive than conventional SCC [ 31 ]. 

Primary intra-osseous SCC is a rare entity and is described as a neoplasm originating primarily within the jawbone without any association with the oral mucosa. Most of them seemingly arise in odontogenic cysts. It shows mandibular and male predilection. Well-to-moderately differentiated SCC is the most common microscopic feature. Surgery or combined surgery and radiation therapy is the treatment of choice [ 9 ]. 

Informed consent was obtained from the patients for publishing their cases and clinical photographs.

## Conclusion

In conclusion, this article presents rare and challenging cases of oral SCC focusing on histopathologic features and differential diagnosis. Familiarity with these characteristics is essential for oral pathologists.
